# Last Trends in Point-of-Care (POC) Diagnostics for the Management of Hematological Indices in Home Care Patients

**DOI:** 10.3390/bios13030345

**Published:** 2023-03-04

**Authors:** Fabrizio Clemente, Amina Antonacci, Maria Teresa Giardi, Valeria Frisulli, Francesco Paolo Tambaro, Viviana Scognamiglio

**Affiliations:** 1 Department of Chemical Sciences and Materials Technologies, Institute of Crystallography (IC-CNR), Via Salaria Km 29.300, 00015 Monterotondo, Italy; 2 Institute of Crystallography (IC-CNR), Department of Chemical Sciences and Materials Technologies, URT Naples c/o Azienda Ospedialiera di Rilievo Nazionale (AORN) Santobono-Pausilipon Via Teresa Ravaschieri 8, 80112 Naples, Italy; 3 Struttura Semplice Dipartimentale Trapianto di Midollo Osseo-Azienda Ospedialiera di Rilievo Nazionale (AORN), Santobono-Pausilipon, 80129 Napoli, Italy

**Keywords:** hemoglobin detection, cancer home care, point of care, telemedicine, commercial devices

## Abstract

Today, complete blood count (CBC) analyses are highly automated and allow for high throughput and accurate and reliable results. However, new analytical tools are in great demand to provide simple, rapid and cost-effective management of hematological indices in home care patients. Chronic disease monitoring at home has become a benefit for patients who are finding cost savings in programs designed to monitor/treat patients in offsite locations. This review reports the latest trends in point-of-care (POC) diagnostics useful for home testing of key hematological counts that may be affected during home therapy treatment.

## 1. Introduction

Over the years, the study of complete blood count (CBC) disorders has served as a paradigm for gaining insight into a variety of diseases. To date, more than 1000 disorders of the synthesis and/or structure of CBC and its concentration in the blood have been identified and characterized. As an example, (i) hemoglobin disorders are associated to sickle cell disease (SCD), beta thalassemia, and diverse hemoglobinopathies; (ii) leukocyte disorders can be linked to inflammatory disease; and (iii) platelet thrombocythemia, reactive thrombocytosis, and thrombocytopenia. On the other hand, marked increases or decreases in blood cell counts could result not only when a genetic disease occurs but also because of treatments for certain diseases, such as cancer. According to the World Health Organization (WHO), the global cancer burden increased in 2018 to 18.1 million new cases, 9.6 million cancer deaths, and 43.8 million people living with cancer [1]. This increase in the number of cancer patients puts enormous pressure on the health system, not just in economic terms. Although hematological malignancies represent the majority of pediatric cancers, central nervous system cancers, neuroblastoma and sarcomas are also represented and all require chemotherapy as a first-line treatment, resulting in bone marrow suppression. If we exclude acute myelogenous leukemias, for which children need to be hospitalized until marrow recovery, almost all other cancers can receive treatment as an outpatient regimen, with periodic cell count monitoring.

Besides cancer, variations in the hematological indices can be strictly associated with several other pathological states, including autoimmune rheumatic diseases, systemic inflammation, arthritis, or SARS-CoV-2 infection among others. In this scenario, changing home care plays a key role in bringing the concept of laboratory settings analysis to routine testing before, during, and after treatment.

The increase in new diagnoses reflects a greater number of people requiring inpatient or outpatient hospitalization. This pressure affects the quality of treatment, the number of patients who can be treated, and the presence and distribution of highly qualified personnel. The COVID-19 pandemic has further exacerbated that burden.

The availability of portable devices as point-of-care (POC) for on-site monitoring has fueled the adoption of out-of-center diagnostics [2,3,4]. The perceived benefits of such home tests are time efficiency, resources, and low costs, which make them a particularly attractive health services for daily home testing, as well as for high-throughput screening of developing countries’ populations with limited access to fully equipped laboratories. Home-based POC diagnostics could not only save valuable time for patients and health care systems, but also protect patients from the risk of inadequate treatment. For this reason, new devices that enable chronic disease patients to monitor and report results to healthcare professionals continue to be developed and adopted by healthcare consumers.

According to a recent report, “The global POC diagnostics market is expected to reach USD 72.0 billion by 2027 from an estimated USD 43.2 billion in 2022, at a CAGR of 10.8% from 2022 to 2027” [5]. The current POC market is dominated by glucose, cardiac, and pregnancy testing, and represents a very rapid-growing segment of a worldwide clinical diagnostic market. The COVID-19 outbreak has further accelerated the global demand for diagnostic kits to rapidly detect the disease [6,7,8]. In recent years, the design of hematological indices in patients under home care has gained momentum. Actually, complete blood counts (CBC) can include many hematological indices, such as mean corpuscular hemoglobin (MCH), lymphocytes (LYM), hematocrit, mean corpuscular hemoglobin concentration (MCHC), hemoglobin (HB), red blood cells (RBC) and subsets, immature granulocytes, immature platelet fraction, and reticulocyte hemoglobin, among others. While conventional methods entail time-consuming and costly laboratory tests and necessitate accompanying the patient to the laboratory for analysis, the latest requirements for home care involve the use of POC diagnostics to evaluate hematological indices through fast, cheap, and disposable tests. These technologies could bring diagnostics to bedside and home testing, minimizing the workload in centralized laboratories. POCs blood tests typically performed during the treatment of diverse diseases are intended for main targets as hemoglobin, leukocytes, hematocrit, and platelet, among others. However, complete blood count (CBC) analysis was not available as a POC test until recently. The newly FDA-approved HemoScreen [9] could fill this gap, being a central example of a small, easy-to-use device that uses disposable cartridges [10]. However, HemoScreen entails laboratory setup instrumentations and is not suitable for home testing.

The following section reports the POC diagnostics available in the literature for the detection of such blood indices, with their pros and cons and integration into telemedicine.

## 2. Point-of-Care Diagnostics for CBC Detection

### 2.1. Point-of-Care Diagnostics for Hemoglobin

Hemoglobin (Hb) monitoring in patients undergoing home therapies is fundamental to increase overall quality of life and to prevent pathologies such as anemia, which may further exacerbate the overall clinical situation. To date, most of the POC solutions available in the literature and on the market aim to quantify hemoglobin in its glycated form [11,12,13,14,15] as a novel tool for diabetes early monitoring in a home setting. The gold standard for Hb quantification in patients is based on diverse methods, such as visual methods (Sahli’s, Dare, Haden, Wintrobe, Tallqvist), spectrophotometric methods (oxyhemoglobin, cyanmethemoglobin, gasometric, automated hemoglobinometry), or other methods (Alkaline-hematin, specific gravity, Lovibond comparator, copper-sulfate, HemoCue and automated hematology analyzers) [16]. These methods, each with its own operating principles, need for a significant amount of blood drawn from the patient and require specialized personnel and high-end instrumentations to be performed. 

The escalation of the COVID-19 pandemic has made such a testing approach impractical, because it requires frequent visits to hospitals or other highly specialized testing facilities. Therefore, with a view to increased home care, novel tools for Hb monitoring that can be used by patients themselves with minimal or no invasive sampling have become very demanding. To this aim, Singh et al. [17] developed an innovative method for spectrophotometric quantification of Hb, which led to an easy implementation in POC devices: a complex matrix was produced employing carbon dots, Nafion and reduced methylene blue, the latter being colorless in reduced form and bright blue in oxidized form. The matrix has a specific redox reaction with Hb present in blood, causing an overall color shift of the solution, which can be quantified using UV–VIS spectroscopy. After the optimization of carbon dots size and exiting light wavelength, the authors assembled a prototype device capable of quantifying Hb in 15 µL of sample, with a limit of detection (LOD) of 1.2 mM and a linear range from 2.4 to 11.2 mM.

**Figure 1:**
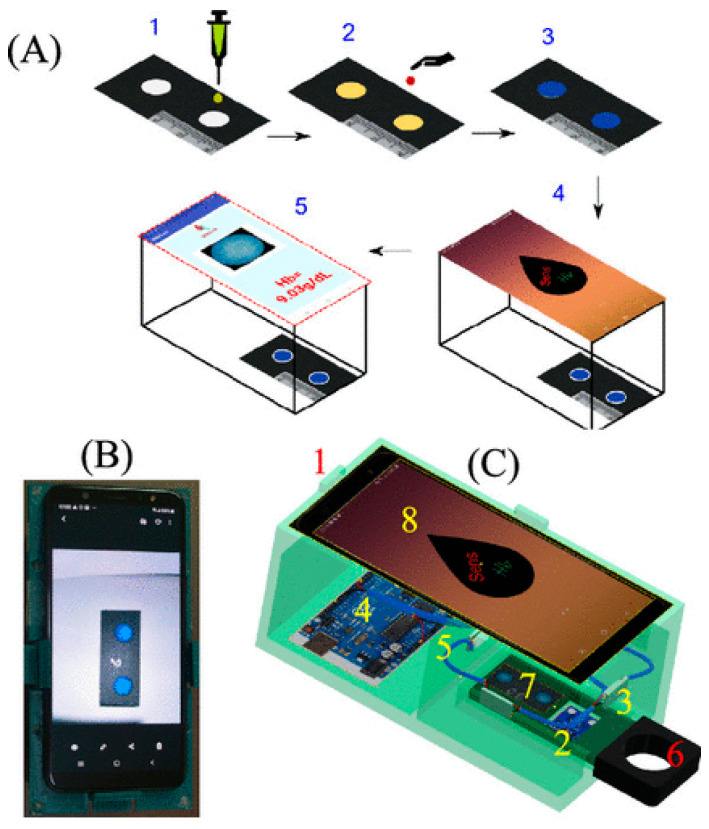
(**A**) Schematic representation of the detection protocol shown stepwise. Five key steps include (1) embedding chemical reagents on the paper device, (2) putting a drop of blood from finger prick, (3) colorimetric changes on the paper device, (4) placement of the device on a cartridge within a customized portable plastic box, and (5) smartphone integration, analytics, and dissemination based on colorimetric signals of the test outcome. For improved performance, prior to step (2), finger-pricked blood is mixed with Drabkin’s solution for 15 s before introducing onto the reaction spots. (**B**) Top-view image of the POC device during the testing of a blood sample. The smartphone app displays two reaction spots of the paper device. A greenish-blue color is developed after the reaction, which is captured by a smartphone. (**C**) Schematic representation of the present device is shown where the smartphone is placed on top of the 3D box. Key components are (1) sliding bars to fit in different models of smartphone, (2) light intensity-measuring microchip that continuously sends feedback to maintain uniform light intensity, (3) LED light source, (4) Arduino board, (5) cable connections between the Arduino board with a light measurement unit and (6) paper-made cartridge, (7) paper-based reaction spots, and (8) smartphone with the Sens-Hb app. Reprinted with permission from [18]. Copyright 2021 American Chemical Society.

A similar principle was developed by Biswas et al. [18]. In this case, 10 µL of blood, obtained by finger pinprick, are mixed with Drabkin’s solution and are then deposited on a cartridge pre-modified with o-tolidine. Hb in blood catalyzes the reaction between Drabkin’s solution and o-tolidine, causing the formation of a greenish-blue product (Figure 1). The color is captured using a standard smartphone camera and is processed by an Android app (named “Sens-Hb”), which analyzes the RGB components of the picture taken and extrapolates the Hb amount in the blood sample. The system was reported to have a very low cost (≈0.02 USD/test), fast response time (total analysis time of 5 min), and a LOD of 2.5 g dL^−1^, with linear response up to 20 g dL^−1^.

Anemia and hemoglobin variant testing together have also become possible due to the system described by An et al. [19], which assembled a system capable of both quantifying the Hb concentration in blood and discriminating between different forms of Hb to identify patients having genetic variants that may lead to sickle cell disease. The system needs 25 µL of blood sample and employs a cartridge in which blood components are electrophoretically separated. The colored bands resulting from the electrophoretic separation are analyzed by a trained artificial neural network, which in a total time of 8 min, extrapolates the Hb amount and forms present in the blood sample. The developed instrument has a linear response range for Hb concentrations from 6.0 to 15.3 g dL^−1^ and a high Pearson correlation coefficient (PCC) of 0.95, *p* < 0.001 compared with the CBC official methodology.

**Figure 2:**
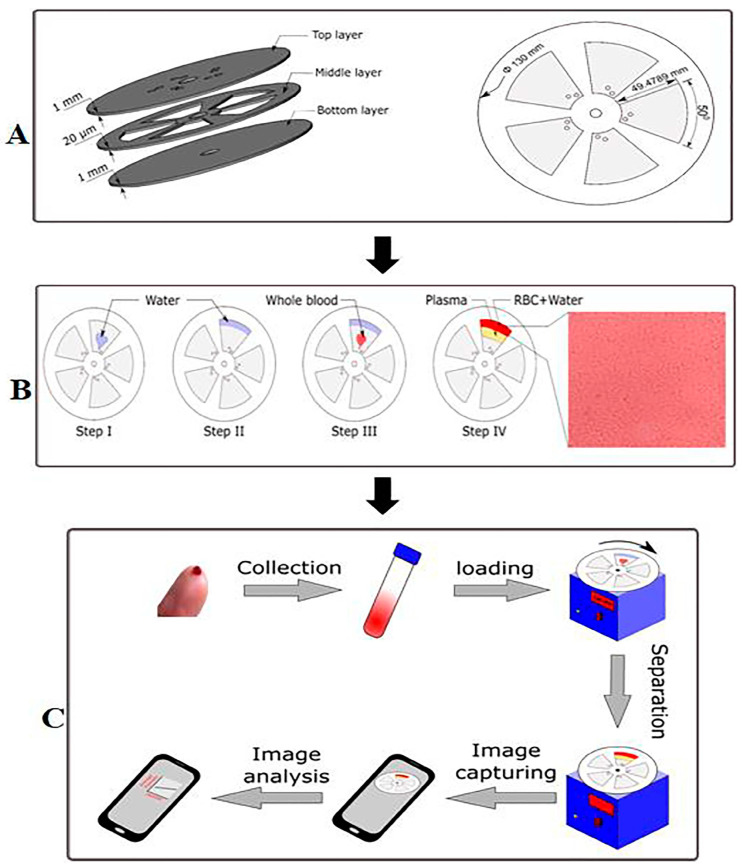
Representation of the experimental process. (**A**) Design and specifications of the compact disc. Side view of the device showing thicknesses of the top, middle and bottom layers which are 1 mm, 20 µm, and 1 mm, respectively. The top view of the compact device showing the channel length which is 49.4789 mm, the angular span of each sector which is 50°, and the diameter of the disc which is 130 mm. (**B**) Stepwise procedure of sample processing in the spinning disc. Loading of DI water in the microchannel (Step I); rotating the disc (at 1000 rpm for 1 min) for transporting water to the outer periphery of the microchannel (Step II); loading of whole blood in the same channel (Step III); rotating the disc (at 2000 rpm for 13 min) to accomplish the separation of RBC from whole blood (Step IV) and mixing with DI water. A magnified view of the RBC lysis in a microchannel is shown as a blown-up figure. (**C**) Schematic illustration of the process flow of Hb estimation in the present POC device. The essential steps include a collection of finger pricked blood into a tube pre-coated with an anti-coagulant, loading of blood in the micro-channel of the disc, separation of RBC due to centrifugation, and mixing with DI water. The rotational speed is displayed using a display unit to attain a precise setting. After osmotic hemolysis leading to the release of Hb, the colored image is captured using a smartphone for rapid analysis. Reprinted with permission from [20]. Copyright 2021 Elsevier.

An interesting, reagent-free tool for Hb quantification was proposed in the work of Chattopadhyay et al. [20]. Here, Hb separation from whole blood is obtained by applying the principles of osmotic lysis and centrifugal separation. In the study, a disk composed of three thin plates and integrating a microfluidic system is described. The disk is divided into sections, each filled with deionized water. A blood sample of 10 µL is collected and added with anticoagulant agent (to prevent occlusion of the microfluidic system) and inserted into the disk (Figure 2). Here, the difference in osmosis forces between blood and deionized water causes hemolysis of the erythrocytes. The disk is then subjected to spinning, making a colored band appear in the external part of the disk, due to hemoglobin accumulation. A picture is taken of the disk after spinning using a common cellphone camera, and the color of the external band is converted to numerical values using an ad hoc developed app, which compared to a calibration curve, gives the hemoglobin concentration. The resulting instrument is reported to have a linear response in the range from 1 to 17 g/dL and is in good agreement with the reference CBC official methodology.

### 2.2. Point-of-Care Diagnostics for Leukocytes

As important immune cells in the human body, white blood cells play a very significant role in the auxiliary diagnosis of many major diseases. Variations of white blood cells number and morphology are predictive of important and serious diseases, with cancer as a crucial example. In the latter case, neutrophil numbers in the blood are markedly abnormal or show signs of immaturity, requiring further blood cell and/or bone marrow examinations. As an example, in patients diagnosed with acute myeloid leukemia, an increase in the number of circulating immature cells in the bone marrow as well as in the proportion of immature cells (i.e., myeloblasts) occur during treatment. For this reason, it is essential to monitor leukocytes during oncological therapies, as they can alter the patient’s physiological state, including the number of lymphocytes and neutrophils. Current methods to measure white blood cells and neutrophil counts, including flow cytometry analysis with laser scattering or impedance detection, scattering of single cells or manual microscopy counts among others, are difficult to perform at the point of care, being cost or labor prohibitive. Trying to overcome this drawback, Majors and colleagues [21] developed an imaging-based system to measure white blood cell and neutrophil counts from a drop of blood called LeukoScope. This device was exploited for the analysis of 136 pediatric subjects at a central hospital in Malawi, with good accuracy on 95.4, 66.7, and 80.0% of samples with normal, low, and high white blood cells counts, and 88.6, 100.0, and 89.3% of samples with normal, low, and high neutrophil counts, respectively. These results highlight that the LeukoScope can help meet the need for POC diagnostics in pediatric patients. However, this instrument, although considered portable by the authors, still has a laboratory setup not suitable for home analysis.

An alternative way of counting the number of blood neutrophils was proposed by Venge and colleagues [22], which described the development of a novel technology for the accurate, rapid, and simplified counting of neutrophils numbers by means of the measurements of one of the major granule constituents in extracted whole blood, myeloperoxidase (MPO). MPO is produced in the bone marrow through the very early stages of development of neutrophils, i.e., myeloblasts. In addition, the concurrent analysis of lactoferrin (LF) in whole blood extracts and the formation of a ratio of MPO to LF could reflect circulating myeloid cell maturity and/or bone marrow activity, since LF is formed in the bone marrow during the later stages of the development of neutrophils, i.e., myelocytes [23]. Due to these results, new rapid and simple point-of-care analytical tools could benefit from this technology, especially when neutrophil counts are associated with inflammatory diseases and blood disorders. To this aim, Barroso et al. [24] exploited the scattering properties of whole blood to determine correlations with red blood cell (RBC) and white blood cell (WBC) counts. In particular, the scattering correction coefficients of visible–nearinfrared (Vis-NIR) of 320–850 nm dog blood absorption spectra were observed to obtain direct correlations of RBC and WBC counts using multivariate linear regression (MLR). This approach allowed for the discrimination of high and low values of both RBC and WBC and their quantification. In detail, a correlation of 0.5739 and a standard error of 1.33 log10 (cells/L) was obtained for RBC, with a correlation of 0.5900 and a standard error of 7.70 log10 (cells/L) for WBC.

In this scenario, it is evident that such systems cannot be considered for point-of-care testing, as they still entail laboratory setup instrumentations and skilled personnel. Despite the efforts made and the new technologies developed, research is still very far from developing POC tests that can be used by patients undergoing home therapy and who need portable, fast and affordable devices to effectively follow variations in leukocyte indices due to diverse therapy. This can be due to several reasons, including the need to process the blood sample before testing. Further research in microfluidics could help overcome this drawback and could enable the design of POC devices for leukocyte analysis, through the use of functional materials (e.g., paper) that allow for separation of blood components without slow and time-consuming pretreatment procedures.

### 2.3. Point-of-Care Diagnostics for Hematocrit

**Figure 3:**
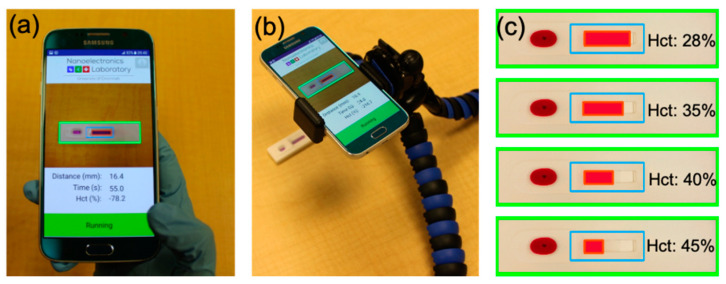
Operation of LFTA on commercial smartphone with fluid flow measurements taken by hand (**a**) and utilizing a smartphone stand (**b**). Illustration of RBC flow distance for different Hct levels after a fixed test time of 150 s (**c**). Reprinted with permission from [25]. Copyright 2020 Elsevier.

Counting blood hematocrit numbers is one of the most common laboratory tests in modern clinical medicine. The hematocrit (Hct), together with the deformability of red blood cells, are the main determinants of the viscosity of whole blood, the alteration of which can be caused by home anticancer therapies that cause related cardiovascular diseases. For this reason, daily hematocrit analysis is crucial to prevent such problems. Frantz et al. [25] presented a rapid POC system based on a smartphone application capable of accurately tracking red blood cell (RBC) flow through a no-reaction lateral flow (nrLFA) assay (Figure 3). Using the smartphone camera, RBC flow is recorded without the need for hardware, and hematocrit is calculated measuring the blood flow distance and time as soon as it is detected in the observation window. This POC system was able to accurately measure (within 1% Hct of nominal values) whole blood Hct in ~10–20 s after sample dispensing.

Hematocrit analysis revealed to be crucial to tackle inaccuracies in glucose measurement, as described by Rao and co-workers [26], who evaluated the accuracy of a glucometer capable of simultaneous measurement of patient’s hematocrit with algorithmic adjustment of glucose results. Venous whole blood samples from healthy volunteers were pooled and reconstituted to produce five different hematocrit (30–60%) concentrations. Each hematocrit specimen was spiked to obtain four glucose concentrations (50–500 mg/dL). This system exhibited good correlation (r = 0.998) with a slope of 0.989 and intercept of 0.827, displaying good agreement with reference methods and existing parameters present on the market, i.e., YSI 2300 whole blood/plasma analyzer (Yellow Springs Instruments, Yellow Springs, OH, USA). Moreover, this system was able not only to measure the hematocrit but also to provide automated correction for the hematocrit effect, thus eliminating the need for a separate hematocrit measurement and saving time.

### 2.4. Point-of-Care Diagnostics for Platelet

**Figure 4:**
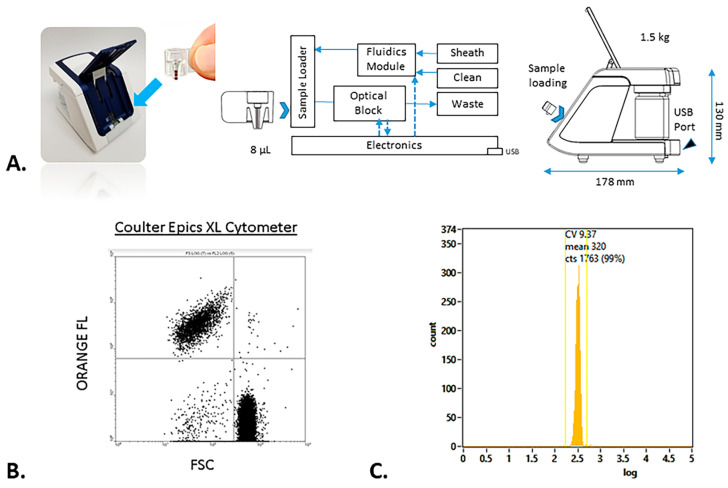
Overview of the rHEALTH method for microvolume cytometry analysis of platelets and data from benchmark cytometer and rHEALTH. (**A**). Left, 8 μL capillary blood sample in a clear plastic consumable is loaded into the instrument for analysis. Middle, The sample consumable is received by a plunger-based, in-line sample loader which seals around its two open ends. Pressure (~70 mbar) is applied to the system which drives the entire sample volume, via hydrodynamic focusing, into a miniaturized optical module for laser-based cytometry detection. A fluidics module with electronic valves manages fluids from the pressurized sheath and clean bottles (60 cc). The analyzed sample passes through the optical block and into the removable waste bottle (60 cc). The electronics with embedded software manage instrument control and data capture. Dashed lines indicate connection to the optical and fluidics modules. Arrows from the electronics module indicate a control function and arrows to the electronics indicate a data function. The USB provides power, control commands, and data output to and from a PC. Right, The device is shown in its side view with dimensions, mass, USB 2.0 port, and sample loading orientation. (**A**). PC computer (not shown) provides power (up to 2.5W) via the USB connection, collects raw data, and performs data analysis. (**B**). XY scattergraphs generated by Coulter cytometer in quadrant analysis. (**C**). rHEALTH histogram analysis of total platelet count includes all orange fluorescent events. Reprinted with permission from [27]. Copyright 2021 Plos.

Complete blood counts, blood film examination, and aggregation are the tests most commonly used to quantify platelets and to determine whether there is thrombocytopenia and/or defective platelet function due to various causes. Conventional assays, with the light transmission aggregometry as the gold standard, are typically not bedside, with detection methods varying from optical to mechanical and electrical transduction. POC testing of platelet count provides real-time data for rapid decision making in many diseases, especially in the case of home cancer treatment. A number of POC platelet function tests have been developed in recent years with different configurations. Several microfluidic devices for whole blood analysis have been also developed, utilizing varied geometries and wetted materials for different purposes. As an example, Dickerson et al. [27] evaluated the accuracy and precision of platelet counting using a new cytometry-based blood analyzer, the rHEALTH ONE (rHEALTH) (Figure 4). This system showed a correlation between capillary and venous blood samples with a slope of 0.9514 and an R^2^ of 0.9684. Moreover, a precision was achieved ranging from 3.1 to 8.0%, in comparison with the International Society of Laboratory Hematology (ISLH) method based on a cytometer/impedance analyzer, which provides a precision of 1.0–10.5%.

Several paper-based microfluidic devices have been also realized for coagulation screening, due to the low cost, hydrophilicity, and ubiquity of paper that is a frontline-used substrate for microfluidics [28]. Li and co-workers [29] described a simple approach for evaluating blood coagulation, developing a microfluidic paper-based lateral flow (LFA) assay for POC self-monitoring screening. This system, based on Millipore C048 cellulose paper, was able to analyze whole blood without any sample pretreatment for the separation of plasma from red blood cells. Blood samples were distributed onto the paper LFA device consisting of a sample pad, an analytical membrane, and a wicking pad. The aqueous component of the plasma is separated from the large blood cells due to the porous nature of the cellulose membrane. As the blood viscosity changes with its ability to clot, the distance RCBs travel in the membrane in a given time may be related to the clotting time of the blood. This distance was found to linearly decrease with a travel rate decreasing from 3.25 to 2.2 mm min^−1^. Compared to conventional plasma clotting analyzers, the described LFA device proved to be much simpler and able to provide a significantly larger linear range of measurement.

## 3. Sample Pretreatment

**Figure 5:**
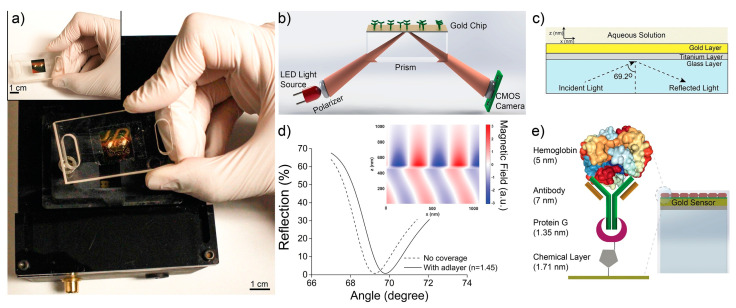
Hand-held plasmonic chip (**a**). The platform consists of a light-emitting diode (LED) to illuminate a cylindrical lens and focuses the light onto a rectangular prism. The reflected light is captured on a CMOS sensor, with the captured image transferred to a computer via the control circuitry and a TV card (**b**). Gold-coated chips fabricated on a glass (Pyrex) wafer (**c**). The binding of a homogenous adlayer (nadlayer = 1.45) with 5 nm of thickness generates a change in resonance angle of the gold chip (**d**). The chip surface is activated with a chemical layer with succinimide ending, protein G layer, and antibody layer to capture hemoglobin molecules (**e**). Reprinted with permission from [30]. Copyright 2020 Elsevier.

Sample pretreatment is a crucial procedure in many biomedical tests for further analysis of blood counts, as this step can efficiently increase the detection limit. With the aim of improving the performance of a biosensor, it is necessary to consider many concerns related to real samples (e.g., blood, serum and plasma), being factors that hinder an efficient analysis of complex matrices, among which the matrix effect, cross-reactivity problems, and the presence of potential interference. Analytical instruments in laboratory settings address these concerns by providing sample pretreatment, with great influence on the quality and quantity of isolated component analysis. These processing methods entail sample collection, cell lysis, the use of anticoagulants (e.g., ethylenediaminetetraacetic acid (EDTA), heparin, citrate), components separation, and storing at various temperatures (4, 22, 37 °C). These procedures should be fast and automatable in the case of POC testing. To this aim, microfluidics demonstrated its capability to avoid sample pretreatment, as these systems are able to separate the blood components enabling the target analytes to reach the detection zone to be analyzed. This can be possible due to the availability of several functional materials (polydimethylsiloxane (PDMS), paper, or Teflon) as well as their modification approaches that can tune the physicochemical features of such materials, including chemical groups, surface charge, hydrophilicity/hydrophobicity, wet strength, and hydrophobic patterns/barriers. To report an example, Inci et al. [30] developed a hand-held, disposable, easy-to-use microfluidic chip able to analyze hemoglobin without sample pretreatment or the introduction of additional reagents after the clinical specimen is loaded. This platform, made with diverse materials such as Pyrex, titanium, and plasmonic gold, consisted of (i) a 705 nm LED with proper lens and polarizer for illuminating the surface, (ii) a glass prism, (iii) a CMOS sensor for measuring the amount of reflection from the surface, and (iv) a TV card for the connection between the platform and computer interface (Figure 5).

**Figure 6:**
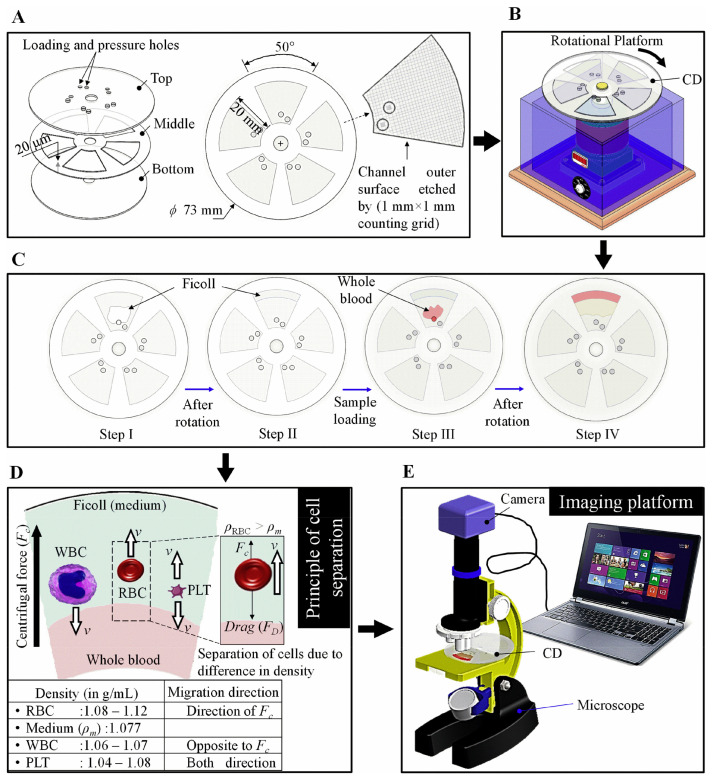
Representation of the experimental process. (**A**) Design specifications of the compact disc, with etched grid for cell counting. (**B**) Spinning platform. (**C**) Stepwise procedure of sample processing in the disc. (**D**) Illustration of the principle of cell separation; transport of cellular components of blood under competing effects of rotational force (centrifugal force), buoyancy force, and drag. (**E**) Light microscope for capturing the images that are then processed in a computer, yielding the counts of various cells based on their sizes along with hematocrit and hemoglobin estimation. Reprinted with permission from [32]. Copyright 2020 Elsevier.

A different microfluidic device was described by Ulum et al. [31] that introduced the use of cotton-thread treatment methods based on EDTA anticoagulant solution for wicking whole blood samples and separating its plasma. This study demonstrated that the deposition of EDTA anticoagulant followed by its drying on the thread at room temperature for 10 s could provide the longest blood wicking with gradual blood plasma separation. This separation occurred through the synergy of cotton fiber, EDTA anticoagulant, and blood platelets, which induce the formation of a fibrin filter via a partial coagulation process in the EDTA-treated microfluidic chip. More recently, Agarwal et al. [32] projected a spinning disc to estimate the content of hematocrit, hemoglobin, RBC, WBC, and platelet with an accuracy of >95%, exploiting the difference in cell densities for their separation into the microfluidic channels (Figure 6). This very simple system enabled multiple sample testing within a single biodegradable disc, eliminating the need for blood downstream processing.

## 4. Integration of Point-of-Care Diagnostics in Telemedicine

Telemedicine can be defined, according to the European Commission, as the provision of healthcare services, through the use of Information and Communication Technology (ICT), in situations where health professionals and the patient are not in the same location. It has become an increasingly valuable and viable method of health service delivery, communication, information transfer, and education. Especially after the COVID-19 emergency, there has been a widespread use of telemedicine for consultations and appointments with clinicians using ICT facilities. These facilities are officially included in many clinical workflow and standards. Particularly, there is a shift to oncologic workflow offering an amount of services in telemedicine (videoconference, teleconsultation, follow up visit, remote monitoring) [33].

Another aspect of telemedicine that is becoming even more established is the integration of home patient monitoring in clinical workflow. Thus, telemedicine can include remote monitoring of parameters useful for follow up such as that from CBC. ICT and medical procedures are available for different homecare monitoring. However, very few examples are available for CBC analysis in oncology. This is because many disease therapies require rigorous measurement and documentations of patient status, and [34] the ability of patient and the clinical reliability of self-reporting in this clinical field is still being debated.

As telemedicine platforms can already be implemented with patient-monitoring systems whose results can be used to intervene in therapy [35], the integration with CBC is poor. In the clinical course of systemic cancer care, the therapies are decided from time to time on CBC evaluation, particularly on white cells or platelet count whose measurement is required before each administration. Auto-diagnosis at home using POC devices as the terminal of larger telemedicine systems is essential in the personalization and humanization of care for these frail patients that move periodically from home to the hospital for single administration. A search on Scopus or Google Scholar for “complete blood counts and telemedicine” reveals very few article in the field (February 2023). Few studies in the literature describe the design of analytical devices for the monitoring of hematological indices assisted by remote sensing [36] or machine learning [37], with future possible integration into telemedicine. However, such systems are not in the form of a POC device, and their real integration into telemedicine is at an early stage. On the other hand, such studies pave the way to a wider use of home screening systems for POC blood testing and telemedicine with lab-on-chip platforms. In this scenario, further research on new reliable, patient-oriented, and specific home care devices based on innovative technologies can solve this lack of solutions [38]. This is because it is emerging that patient remote monitoring in oncology still requires the intervention of medical doctors and the carrying out of more reliable clinical investigations.

## 5. Commercial CBC Count at Home

CBC count devices for POC analysis are only recently gaining traction as market products. This may be correlated to different factors, because a simple blood test, which can be performed even in small testing labs, gives complete, precise and detailed results concerning blood composition, even regarding the different types of cells present (number of erythrocytes, number and type of leukocytes, platelets count and various minor compound presence and quantification).

Recently, with the rise of the COVID-19 pandemic, the necessity to minimize the crowding of people who, undergoing their therapies, may be in a state of weakened immune system, making them prone to severe COVID-19 forms, has emerged. On the other hand, treatment of several diseases, including cancer, often has a severely negative impact on bone marrow cell production, resulting in patients suffering from pathologies attributable to severe anemia, an overall weakened immune system, and reduced coagulation factors due to reduced amounts of platelets in the blood (thrombocytopenia). These two factors have pushed for the development of systems allowing patients under home care to monitor important parameters of their blood composition, such as hemoglobin concentration, number and type of leukocytes, platelets concentration, and so on.

POC instrumentation for the quantification of hemoglobin seems to be focused on two main methods of signal transduction: optical and electrochemical. Three out of the four systems reported in Table 1 use optical transduction, however with significantly different signal processing. Two systems (HemoCue Hb 801 System and DiaSpect Tm) perform a direct spectroscopic quantification of the hemoglobin present in a blood sample, without the need for additional reagents. The third system (HemoScreen Hematology Analyzer) employs viscoelastic focusing on the blood sample to align all blood components on a single plane. Then, an image is captured and processed using machine vision algorithms, providing an accurate determination of various blood parameters, including hemoglobin concentration, which is calculated based on the optical density measured on individual intact cells.

This demonstrates that a number of instruments are reaching the market, but most of them are not yet in the form of a home test because they still do not own crucial features such as portability, simplicity, and accuracy for bedside analysis. Such systems have serious flaws in terms of speed, timeliness, communication of results, and cannot be handled by non-laboratory personnel, thus showing multiple challenges that constrain their market penetration. For these reasons, further research is required to provide vanguard technologies with increased innate robustness and better diagnostic performance to become benchmark products with a better probability of penetrating the market.

## 6. Conclusions and Future Perspectives

Through the technological advances of the last decade, the process of diagnosing hematological indices is gradually entering a new era in the field of diagnostic medicine. However, the current healthcare laboratory system and the resistance to the penetration of molecular diagnostics challenge the need for speed in diagnosis. In the context where the health of a patient in home care is highly dependent on obtaining a diagnostic response within 6 h, ideally less than 1 h, molecular diagnostic testing capability at the point of care is required. In this scenario, POC diagnostics are becoming a manifest reality due to timely results, ease of use, low cost, and portability, with an evident improvement in the health service. Although POC testing can be performed in specialized laboratories or in hospitals, many challenges still need to be overcome. Due to the convergence of crosscutting technologies such as nanotechnology, microfluidics, and materials science, we expect the obtainment of further knowledge for the design of real POC tests as well as their entrance into integrated disposable devices for bedside and home use to occur within a few years.

## Figures and Tables

**Table 1:** On-the-market products for hemoglobin quantification in POC settings.

Test	Manufacturer	Type of Test	Measurement Range	Volume Sample Needed for the Test	FDA Approval	Point of Application	LINK FDA/Manufacturer Site
HemoCue Hb 801 System	HemoCue AB	Spectrophotometric Optical absorbance	1–25.6 g/dL	10 μL	Yes	Professional in vitro diagnostic	https://www.accessdata.fda.gov/cdrh_docs/reviews/K181751.pdf accessed on 1 March 2023
DiaSpect Tm	EKF-diagnostic GmbH	Spectrophotometric Optical absorbance	1.2–25.5 g/dL	10 μL	Yes	Under prescription order	https://www.accessdata.fda.gov/cdrh_docs/reviews/K172173.pdf accessed on 1 March 2023
StatStrip Hb/Hct	Nova Biomedica	Electrochemistry	6.5–22 g/dL	1.6 μL	No		https://novabiomedical.com/statstrip-lac-hb-hct/ accessed on 1 March 2023
HemoScreen Hematology Analyzer	PixCell Medical Technologies Ltd.	Viscoelastic focusing coupled with high-res imaging and machine vision algorithms	3.94–24.20 g/dL	40 μL	Yes	Clinical laboratorial and POC settings	https://www.accessdata.fda.gov/cdrh_docs/reviews/K180020.pdf accessed on 1 March 2023
GTT2/3	Thromboquest Limited, UK	The instrument detects the time interval between two consecutive blood drops falling into the reservoir	-	4 mL	No	POC, requires specialist	https://www.thromboquest.com/newpage accessed on 1 March 2023
AGGRO/LINK^®^ Opti8	Chrono-log Corporation	Optical Aggregation and Ristocetin CoFactor Testing	-	250–500 µL	No	Requires specialist	http://www.chronolog.com/Model490_4-4.html accessed on 1 March 2023

A fourth system employs electrochemical transduction to calculate the hemoglobin concentration in human blood: StatStrip Hb/Hct.

## Data Availability

As this is a review article, no data are present.
